# Interplay between Virus-Specific Effector Response and Foxp3^+^ Regulatory T Cells in Measles Virus Immunopathogenesis

**DOI:** 10.1371/journal.pone.0004948

**Published:** 2009-03-25

**Authors:** Caroline I. Sellin, Jean-François Jégou, Joëlle Renneson, Johan Druelle, T. Fabian Wild, Julien C. Marie, Branka Horvat

**Affiliations:** 1 Immunobiology of Viral Infections, Inserm, U758, Lyon, France; 2 Ecole Normale Supérieure de Lyon, Lyon, France; 3 IFR128 BioSciences Lyon-Gerland Lyon-Sud, Lyon, France; 4 Université Lyon 1, Lyon, France; New York University School of Medicine, United States of America

## Abstract

Measles is a highly contagious childhood disease associated with an immunological paradox: although a strong virus-specific immune response results in virus clearance and the establishment of a life-long immunity, measles infection is followed by an acute and profound immunosuppression leading to an increased susceptibility to secondary infections and high infant mortality. In certain cases, measles is followed by fatal neurological complications. To elucidate measles immunopathology, we have analyzed the immune response to measles virus in mice transgenic for the measles virus receptor, human CD150. These animals are highly susceptible to intranasal infection with wild-type measles strains. Similarly to what has been observed in children with measles, infection of suckling transgenic mice leads to a robust activation of both T and B lymphocytes, generation of virus-specific cytotoxic T cells and antibody responses. Interestingly, Foxp3^+^CD25^+^CD4^+^ regulatory T cells are highly enriched following infection, both in the periphery and in the brain, where the virus intensively replicates. Although specific anti-viral responses develop in spite of increased frequency of regulatory T cells, the capability of T lymphocytes to respond to virus-unrelated antigens was strongly suppressed. Infected adult CD150 transgenic mice crossed in an interferon receptor type I-deficient background develop generalized immunosuppression with an increased frequency of CD4^+^CD25^+^Foxp3^+^ T cells and strong reduction of the hypersensitivity response. These results show that measles virus affects regulatory T-cell homeostasis and suggest that an interplay between virus-specific effector responses and regulatory T cells plays an important role in measles immunopathogenesis. A better understanding of the balance between measles-induced effector and regulatory T cells, both in the periphery and in the brain, may be of critical importance in the design of novel approaches for the prevention and treatment of measles pathology.

## Introduction

Measles is a highly contagious childhood disease resulting in an acute respiratory infection, followed in certain cases by fatal neurological complications. Measles Virus (MV) remains a major cause of childhood morbidity and mortality in developing countries and measles outbreaks occur regularly in industrialized countries [1]. MV infection induces an efficient immune response, leading to viral clearance and a life-long immunity against re-infections [2], [3]. In addition, MV infection gives rise to a non-specific activation of the immune system characterized by a spontaneous proliferation of peripheral blood mononuclear cells and an up-regulation of activation-associated cell-surface markers [4], [5]. Along with this immune activation, MV induces a transient but severe immunosuppression, which increases the susceptibility of patients with measles to secondary bacterial and viral infections, leading to high infant morbidity and mortality. Immune abnormalities include the disappearance of the delayed type of hypersensitivity responses [6], [7], an impaired proliferation of peripheral blood lymphocytes [8] as well as allospecific cytotoxicity [9]. In experimentally infected monkeys, both activation of the immune response and immunosuppression have been observed [10]. However, the immunological mechanism responsible for this apparent measles paradox remains elusive.

Measles was the first disease recognized to be a cause of virus-induced immunodeficiency [6]. Multiple mechanisms have been advocated to explain this immunosuppression. Type 2 polarization of cytokine responses occurs during the late stages of measles with an increase in the secretion of interleukin 4 (IL-4) and a decrease of IL-2 and interferon γ (IFN-γ) levels [11]. The production of the pro-inflammatory cytokine IL-12 is also markedly suppressed in patients with measles [12] and the anti-inflammatory cytokine IL-10 increased [13], [14]. Furthermore, the importance of different MV proteins in the induction of immunosuppression has been demonstrated [15]. MV glycoproteins, hemagglutinin (H) and fusion protein (F) could induce a surface-contact-mediated signaling, leading to the disruption of Akt kinase activation and inhibition of cell proliferation [16]. Moreover, the interaction of MV nucleoprotein with Fcγ receptor on antigen-presenting cells is implicated in the suppression of cell-mediated responses, [17], [18], [19] and in the induction of the T regulatory immune response, following a chronic exposure [20].

The generation of T cell immunity is regulated by multiple cellular and molecular events. During the past years, the role of Foxp3-expressing CD4^+^ T regulatory cells (Tregs) has become more evident, not only in the prevention of autoimmunity, but also in the control of antimicrobial immune responses, especially against pathogens that induce a persistent infection [21]. However, the influence of CD4^+^Foxp3^+^ Tregs in response to acute virus infection is largely unknown. CD4^+^CD25^+^ T cells were recently shown to be increased in adult measles patients, suggesting their potential role during infection [13], [14], although this finding has been contradicted by the others [22]. We have, therefore, analyzed the immunopathogenesis of an acute MV infection in mice transgenic for the human CD150 molecule, a receptor for both vaccine and wild-type MV strains [23]. Suckling CD150 transgenic mice are highly susceptible to intranasal MV infection and develop clinical signs of neurological disease shortly after the infection [24]. We show here, that following its natural route of infection, wild-type MV induces a strong and broad activation of the immune system, the generation of MV-specific humoral and cellular anti-viral responses, accompanied by an increase in the frequency of regulatory CD4^+^CD25^+^Foxp3^+^ T cells. Although their suppressive function was not altered *in vitro*, the increased frequency of Tregs observed after infection correlated with the significant suppression of T cell response in mixed leukocyte reaction. Moreover, we show that adult CD150 transgenic mice, crossed into IFN α/β receptor deficient background, are highly susceptible to MV infection and develop generalized immunosuppression characterized by an increased frequency of CD4^+^CD25^+^Foxp3^+^ T cells and strong reduction of hypersensitivity response. These results demonstrate the ability of an acute MV infection to affect Foxp3^+^ Treg homeostasis and shed a new light on the immunological basis of measles paradox, where the strong anti-viral reaction is associated with a profound suppression of responses to unrelated antigens.

## Results

### Measles virus infection induces a strong activation of the immune system in CD150 transgenic mice

**Figure 1:**
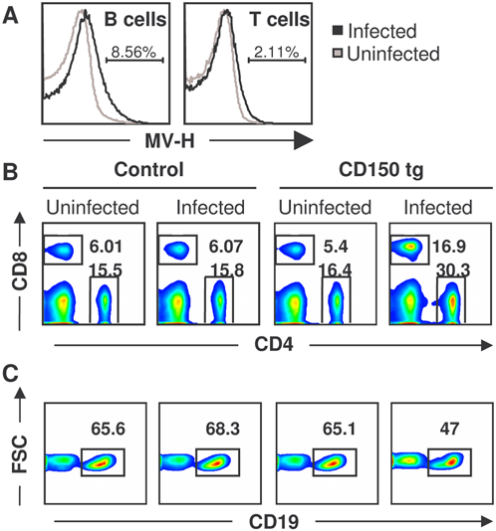
MV infection induces a strong activation of the immune response in CD150 transgenic mice. CD150 transgenic mice and nontransgenic littermates (control) were inoculated i.n. with MV or medium (uninfected). Spleens were harvested 13 dpi and cells were analyzed by flow cytometry. (A) Expression of MV hemagglutinin (H) antigen on the surface of spleen cells from uninfected (grey line) and MV-infected (black line) CD150 transgenic mice, gated on CD19^+^ (B cells) or CD3^+^ (T cells) cells. Numbers are percentages of cells expressing MV H antigen in infected conditions. (B) Staining for CD4^+^ and CD8^+^ T cells and (C) CD19^+^ B cells, among analyzed splenocytes. Results are representative of 8 different experiments, each involving 3–6 mice. Differences between CD150 transgenic infected mice and the other groups were statistically significant (p<0.05, Student t-test).

We have generated transgenic mice expressing the human MV receptor CD150, which are highly sensitive to MV infection. In contrast to nontransgenic mice, intranasal inoculation of suckling CD150 transgenic mice with wild-type MV strains induces an acute neurological syndrome, followed with high mortality [24]. Here, we analyzed the immune response in these mice after MV infection. Following intranasal infection, the expression of MV hemagglutinin was detected on both T and B lymphocytes, only in infected CD150 transgenic mice (Fig. 1A). Similarly to the severe lymphopenia observed in children [3], [25], MV infection of suckling CD150 transgenic mice greatly reduced the number of lymphoid cells: the number of splenocytes 10–14 days after infection (dpi) was regularly lower in infected transgenic mice (4.7±2.1×10^7^) than in infected nontransgenic littermates (9.2±3.6×10^7^) (for 15 and 12 mice respectively, p<0.05, student t-test). The additional changes in the spleen T cell-compartment of the immune system included a two-fold increase in the percentage of CD4^+^ T cells and a three-fold increase in CD8^+^ T cell percentage in infected transgenic mice, in comparison to the all other 3 groups analyzed (Fig. 1B, Table 1). The greatest increase in the percentage of T cells in the spleen was found in mice with the most severe clinical symptoms (weight loss, ataxia, seizures). In contrast, a moderate but reproducible decrease in the percentage of B cells was observed (−30% as compared to controls) (Fig. 1C).

**Table 1:** Measles Virus-induced activation of lymphocytes in CD150 transgenic mice.

Percentage of positive cells*		Control		CD150 tg	
		Uninfected	Infected	Uninfected	Infected
CD4^+^		13,33±2,31	13,75±1,81	15,56±2,46	34,36±14,71**
CD4^+^	CD69^+^	2,77±0,44	3,51±0,40	3,37±0,76	8,93±2,39***
CD4^+^	CD62L^+^	86,55±2,57	83,63±1,45	83,60±2,89	71,42±11,42
CD4^+^	CD44^high^	9,57±1,37	9,05±0,93	7,27±0,31	8,67±0,88
CD4^+^	CD25^+^Foxp3^+^	7,27±0,78	7,08±0,38	7,27±0,35	10,02±1,20***
CD8^+^		4,74±0,79	4,58±1,26	6,24±1,86	13,95±5,14**
CD8^+^	CD69^+^	2,08±0,46	2,53±0,42	2,90±1,10	10,05±6,87**
CD8^+^	CD62L^+^	76,88±4,15	83,18±2,17	73,78±3,84	60,54±8,24**
CD8^+^	CD44^high^	16,30±1,08	16,27±2,06	15,73±3,09	9,59±5,95
CD19^+^		63,07±4,24	71,95±4,20	53,58±13,64	38,02±16,71
CD19^+^	CD69^+^	1,83±0,22	2,11±0,23	2,33±0,92	9,77±5,60**
CD19^+^	CD80^+^	1,43±0,13	1,46±0,56	1,20±0,15	2,77±0,83

* Mice were inoculated intranasally with either MV (500 to 1000 PFU) or with medium (uninfected). Splenocytes were prepared 13 days post-infection, stained with indicated antibodies and analyzed by flow cytometry. Results are expressed as the mean percentage (+/−SD) of positive cells (CD69^+^, CD62L^+^, CD44^high^ or CD80^+^) in a given cell subset (CD4^+^, CD8^+^ or CD19^+^) (4 to 6 mice per group). These results are from one experiment representative out of three.

** p<0.05.

*** p<0.01, Student t-test, calculated between CD150 infected and noninfected mice.

Numerous studies have reported that MV infection in humans strongly activates the immune system, including both T [4], [5], [26] and B lymphocytes [27]. We therefore analyzed different activation markers on splenocytes from MV-infected transgenic mice and observed highly activated phenotypes of both T and B lymphocytes. The proportion of CD69^+^ cells in the CD4^+^T cell subset was increased three-fold in infected CD150 transgenic mice (Table 1). The activation phenotype was even more pronounced in the CD8^+^ T cell subset, with a four-fold increase in the expression of activation-associated marker CD69. This was associated with a significant decrease in CD62L expression, a cell surface molecule implicated in lymphocytes homing to peripheral tissue, suggesting the migration of lymphocytes from the spleen to the periphery. Finally, B cells, although less numerous, were strongly activated in infected CD150 transgenic mice based on increased CD69 and CD80 expression (Table 1).

### MV-infected CD150 transgenic mice develop a specific humoral and cellular response

**Figure 2:**
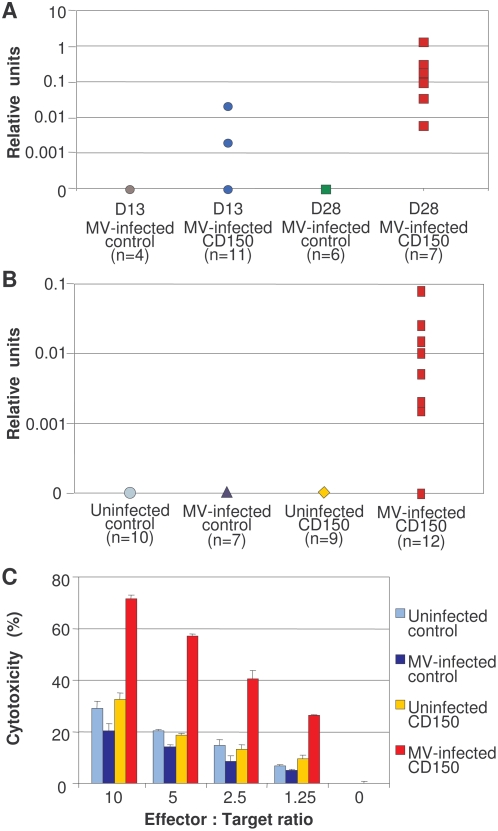
MV infection induces a specific antibody and cytotoxic response in CD150 transgenic mice. (A, B) Production of anti-MV nucleoprotein (N) antibodies (IgG) was measured in serum of individual mice by ELISA and the number of tested animals is indicated in parenthesis. Titers are expressed as relative units. (A) Mice were immunized with low titre of MV (200 PFU) and serum was collected on days 13 and 28, or (B) mice were immunized with higher dose of MV (10^3^ PFU) and serum was collected on day 13 post infection. (C) To analyze cellular anti-viral response, splenocytes were harvested and restimulated with target cells expressing MV N gene (P815-N) for one week (3 to 8 pooled mice per group). Cytotoxic activity was measured as described in Methods; results are expressed as the mean percentage of N-specific cytotoxic activity from duplicate cultures (+/−SD) and the data are from one representative experiment out of three. Cytotoxic activity of lymphocytes obtained from MV-infected CD150 mice was significantly higher compared to the other groups, (p<0.05, Mann-Whitney U test).

The important activation phenotype of B and T lymphocytes, observed in MV-infected CD150 transgenic mice, prompted us to analyze anti-MV antibody production and cytotoxic activity in these animals. When mice were infected with a low dose of virus (200 pfu) to reduce the mortality observed in this model [24], all mice developed N-specific antibodies by one month post-infection and the majority of sera (5 out of 7 sera tested) had high neutralization titers (Fig 2A). MV intra-nasal infection with the high dose of the virus (10^3^ PFU) induced the generation of nucleoprotein (N)-specific IgG antibodies in 60% of infected CD150 transgenic mice at 13 dpi (Fig. 2B). Furthermore, a strong N-specific cytotoxic activity was detected in infected CD150 transgenic mice at 13 dpi, showing that these mice developed a specific cellular anti-measles response (Fig. 2B). However, differences in the IFN-γ production were not observed (data not shown). Thus, as observed in patients with measles, who developed both neutralizing antibody and T cell specific responses [2], [3], infected CD150 transgenic mice are capable of mounting both MV-specific humoral and cellular immune responses.

### Enrichment of the Foxp3^+^ regulatory T cell population after MV infection

**Figure 3:**
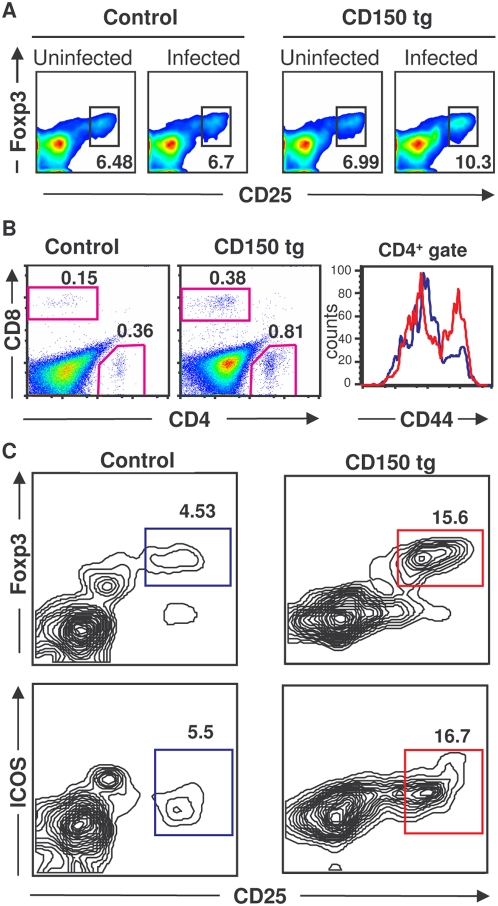
MV infection increases the proportion of CD4^+^CD25^+^Foxp3^+^ Tregs. (A) Splenocytes from CD150 or nontransgenic mice (control), inoculated i.n. with either MV or medium (uninfected), were harvested 13 dpi and stained for CD4 and CD25 followed by anti-Foxp3 intracellular staining and analyzed by flow cytometry. (B, C) CD150×Foxp3-GFP transgenic mice and Foxp3-GFP littermates (control) were inoculated i.n. with MV. Brains were harvested 8 dpi and analyzed by flow cytometry as described in Methods. (B) Proportion of infiltrating CD4^+^ and CD8^+^ T lymphocytes in the brain (two left panels); expression of the CD44 activation marker on CD4^+^ T lymphocytes (right panel, CD150 transgenic in red and nontransgenic control in blue). (C) Tregs detected by the co-expression of Foxp3 and CD25 or ICOS. Results are representative of 4 independent experiments, each involving 4–7 mice per group. Differences between infected and noninfecetd mice were statistically significant (p<0.05, Student t-test).

The intensive immune activation observed both in measles patients and infected CD150 transgenic mice may need to be controlled by Treg cells to avoid tissue damage. This cell population have been shown to regulate the outcome of the infection having either beneficial or detrimental role for the host [21], [28]. We therefore studied the Treg cell population (CD4^+^CD25^high^Foxp3^+^) in MV-infected CD150 mice. Interestingly, we found a significant increase in the percentage of Tregs in the spleen of infected transgenic mice (Fig. 3A). These CD4^+^CD25^high^Foxp3^+^ cells presented a conventional phenotype, based on the expression of CD69, CD62L and GITR, but we did not detect IL-10 production (data not shown).

**Figure 4:**
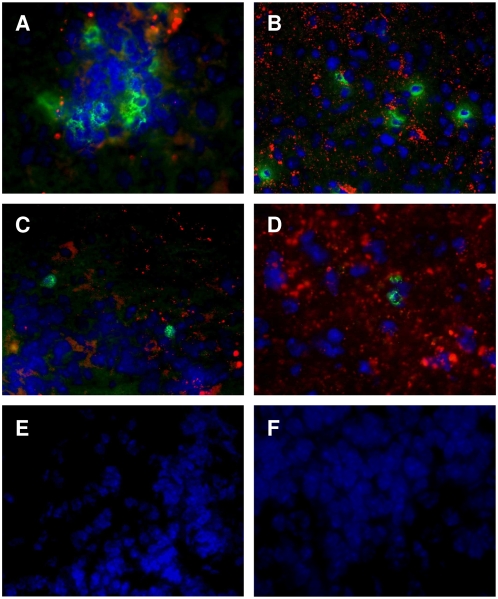
T lymphocyte infiltration at the sites of MV-brain infection. Brain sections from suckling CD150-transgenic (A–E) and nontransgenic littermate mice (F) infected with MV were analyzed by immunohistofluorescence for MV nucleoprotein (N) localization (A–F, in red) and the presence of CD4^+^ (A, in green), CD8^+^ (B, in green) and Foxp3^+^ T cells (C–F, in green). Cell nuclei were counterstained with DAPI (in blue). Infiltrating T CD4^+^ and CD8^+^ lymphocytes were detected in brains from CD150 transgenic mice (A and B, respectively) at the sites of MV infection identified by a N-specific labelling (red dots) but not in their nontransgenic littermates (not shown). Images are shown at 40× original magnification and are representative of three to five mice per group.

MV-induced brain infection is a serious complication of measles and altered immune response may be implicated in this MV-induced neuropathology. Therefore, we analyzed the frequency of regulatory T cells (Foxp3^+^ CD25^high^ CD4^+^) in the brain of MV-infected mice, where the virus intensively replicates [24]. We crossed CD150 transgenic mice into Foxp3-GFP background [29] to facilitate the follow-up of Foxp3^+^ Tregs. Although T lymphocytes represented a minority of the harvested cells from the brain, the percentage of both CD4^+^ and CD8^+^ T lymphocytes was two-fold increased in infected transgenic brains (Fig. 3B). Infiltrating CD4^+^ lymphocytes presented an activated phenotype, expressing a high level of the CD44 marker (Fig. 2B), suggesting their recent migration to brain tissue. Moreover, among CD4^+^ cells, a high enrichment (three fold) of CD25^high^ Foxp3^+^ T lymphocytes was observed (Fig. 3C). These CD25^+^ Foxp3^+^ T lymphocytes expressed the Treg markers ICOS (Fig. 3C) and GITR (data not shown). In addition, immunohistofluorescence experiments were carried out to analyze the localization of regulatory T cells in infected brain parenchyma. In MV-infected CD150-transgenic mice, both infiltrating CD4^+^ and CD8^+^ T cells (Fig. 4A and 4B, respectively) were found in infected brain regions revealed by a MV N-specific staining. These regions included mainly olfactory bulbs and nuclei, hypothalamus and, at a lesser extent, the midbrain, the brainstem and/or periventricular spaces (not shown). In agreement with previous observations that Tregs are attracted to the site of inflammation [21], we have detected Foxp3^+^ Treg cells in the brain at the sites of MV infection (Fig. 4C and 4D). Neither MV infection nor infiltrating Treg cells were observed in brains from infected wild-type mice (Fig. 4E) or uninfected CD150-transgenic mice (Fig 4F). Together, these results demonstrate that Tregs accumulate in the brain in the areas of MV infection.

### Characterization of the Treg cell function

**Figure 5:**
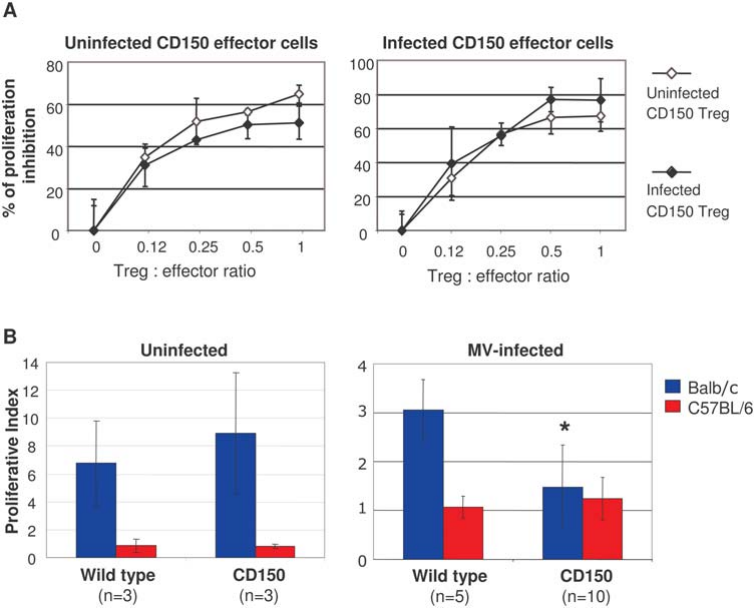
Characterization of Treg function following MV infection. (A) Analysis of suppressor activity of Tregs isolated from CD150 mice, inoculated with MV (open symbol) or with medium (full symbol), in cocultures with CD4^+^CD25^−^ effector T cells from either uninfected CD150 mice (left panel), or infected CD150 mice (right panel) in the presence of irradiated CD4^+^ T cell–depleted splenic APCs and Con A (3 to 5 pooled mice per group). Proliferation of Tregs from either control or CD150 infected mice in response to Con A was ∼300–600 cpm. The results are shown as the mean percentage of proliferation inhibition in triplicate cultures±SD. Results are representative of three different experiments. (B) Splenocytes isolated from either uninfected (left panel) or MV-infected (right panel) CD150 mice and their nontransgenic littermates (wild type) were stimulated with either irradiated Balb/c or C57Bl/6 splenocytes in MLR in triplicate cultures, as described in Methods. Proliferation is expressed as mean proliferation index±SD and is representative from two independent experiments (* P<0,01, Student t-test).

To analyze the Treg suppressor activity, purified CD25^+^CD4^+^ T cells from infected and non-infected CD150 transgenic mice were co-cultured with CD25^−^CD4^+^ effector T cells in the presence of irradiated CD4^+^ T cell-depleted antigen presenting cells (APC) and concanavalin A (ConA). No significant difference was observed in suppressor activity between MV-infected and non-infected transgenic Tregs on proliferation of effector cells, purified from either uninfected or infected CD150 transgenic mice (Fig. 5A). Thus, MV-infection does not seem to modulate the regulatory function of Tregs. Although the suppressive capacity of Tregs in vitro is not changed, their increased frequency in vivo may induce the immunomodulatory effects in MV infection.

We next analyzed the ability of T lymphocytes isolated from either MV-infected or noninfected CD150 mice to respond to MV-unrelated alloantigens. We tested the ability of T lymphocytes to proliferate in a mixed leukocyte reaction (MLR), a prototype of T cell response against virus-unrelated antigens, shown to be under the control of regulatory T cells [30]. Lymphocytes from uninfected CD150 and wild type mice, both in C57Bl/6 background, responded similarly in MLR against irradiated Balb/c splenocytes (Fig. 5B). Strikingly, T lymphocytes harvested from MV-infected transgenic mice were highly impaired in their response to alloantigens presented by Balb/c APC, compared with non-transgenic littermates (Fig. 5B). These results suggest that MV infection suppresses ex vivo T cell responses against virus-unrelated antigens.

**Figure 6:**
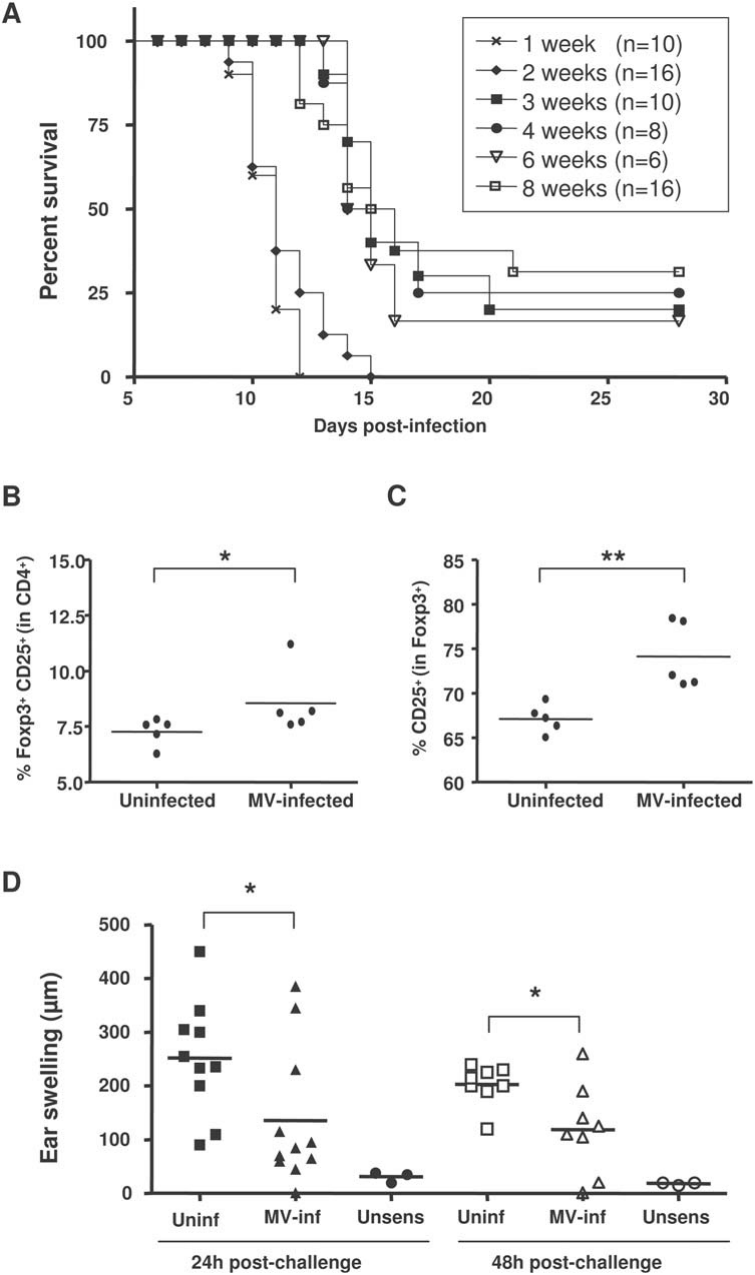
MV-induced immunosuppression in adult transgenic mice. (A) Transgenic CD150×IFNα/βR KO mice were infected i.n. with MV at different ages and monitored for survival by Kaplan-Meier analysis. (B, C) Groups of 5 mice, 6 to 7 week-old, were infected i.n. with MV or left untreated. Splenocytes were harvested at 11 dpi and stained for CD4 and CD25 followed by anti-Foxp3 intracellular staining and analyzed by flow cytometry. Results are presented: (B) as the percentage of CD25^+^Foxp3^+^ cells within CD4^+^ compartment, for each analyzed animal and (C) as a percentage of CD25^+^ cells within the CD4^+^Foxp3^+^ population. Horizontal bars correspond to mean values. (D) Groups of 10 mice, 6 to 7 weeks old, were infected i.n. with MV or left untreated. Seven days later, mice were sensitized with DNFB 0.5% on the ventral skin or left unsensibilized (unsens). All mice were challenged 5 days later with DNFB 0.1% on the left ear. Results are presented as the individual ear swelling of two independent experiments and horizontal bars correspond to mean values. (** p<0.01, * p<0.05, Mann-Whitney test).

The hallmark of MV-induced immunosuppression in children is the strong inhibition of delayed type hypersensitivity responses. Since it is possible to test this type of cellular immune response only in adult mice, and as only suckling CD150 mice are susceptible to MV infection, we then crossed these mice into the IFN receptor type 1 deficient background and analyzed their susceptibility to intranasal MV infection at different ages. Similarly to the other transgenic models for MV infection [31] in the absence of IFN type 1 signaling, mice remain highly susceptible to MV at an adult age, providing an additional model to analyze MV-induced immunomodulation (Fig. 6A). We have, therefore, further analyzed the immunopathogenesis of MV infection in 6 week-old mice. In contrast to suckling mice, the proportion of CD4^+^ and CD8^+^ lymphocytes did not change in adult mice (data not shown). However, the percentage of CD4^+^CD25^+^Foxp3^+^ T cells increased significantly after the infection (Fig. 6B), following the same pattern seen in suckling mice (Fig. 3A). Furthermore, these Foxp3^+^ cells expressed significantly higher level of the CD25 marker in infected mice (Fig. 6C). We then analyzed the capacity of these mice to generate contact hypersensitivity response to the hapten 1-fluoro-2,4-dinitrobenzene (DNFB) following MV infection. Interestingly, the contact hypersensitivity response was greatly reduced in MV-infected mice, up to 48 h after sensibilization (Fig. 6D), demonstrating the MV-induced inhibition of cellular immune responses in vivo. Thus, MV infection in this transgenic model induces a severe suppression of immune responses to MV-unrelated antigens, associated with an increased frequency of Tregs.

## Discussion

The salient features of measles infections are characterized by a robust activation of the immune system and the generation of anti-viral responses, followed by a transient but profound immunosuppression to virus-nonrelated immune reaction [2], [3]. In this study we have analyzed the immunopathogenesis of measles in transgenic mice expressing the human CD150 receptor. Similarly to what has been observed in patients with measles, we show that MV infection in these transgenic mice leads to the activation of the immune system and the generation of effector cells in parallel to the suppression of virus-unrelated T cell responses, including the hypersensitivity reaction. Moreover, the increased frequency of Foxp3^+^ Tregs was observed in both suckling and adult infected transgenic mice. Although MV infection could induce different and nonexclusive mechanisms that may contribute to the generalized immunosuppression, the modulation of Foxp3^+^ Treg homeostasis, may be the essential step in measles immunopathogenesis. An important role of Tregs was demonstrated in different viral infections, where the outcome of an infection has been shown to depend on the balance between T regulatory and effector immune functions [28]. In chronic hepatitis C virus infection in humans an excess number or function of Tregs can inhibit effector immune responses and thus allow pathogen long-term persistence, up to host cell destruction, leading sometimes to massive liver damage [32]. In contrast, the low frequency of Tregs in severe forms of an acute dengue virus infection in humans seems to be insufficient to circumvent effector functions and the development of immunopathology [33]. In measles, the interplay between effector and regulatory response, shown in this study, could be critical in the immunopathogenesis of this infectious disease and the adequate balance between these two arms of immunity may have an essential role in the outcome of the infection.

Virus may increase the frequency of host Treg cells by different mechanisms, including their expansion, longer survival, conversion, higher recruitment or retention at the site of infection or in the periphery and may involve either direct mechanisms via cell-cell contact or production of inhibitory cytokines such as IL-10 or TGF-ß [28]. Although the MV-induced increase of the Treg frequency was evident both in the brain and in periphery, the mechanisms still remain to be defined. Interferon type 1 does not seem to be critical, as mice deficient for this cytokine were strongly immunosuppressed after MV infection in our study. In addition, the higher expression of CD25 on CD4^+^Foxp3^+^ Tregs, seen in this study, reveals their activated phenotype, potentially associated with cell expansion. Several reports have suggested the implication of dendritic cells (DC) in MV-induced immunosuppression [34], [35], [36]. DC play an important role in the induction of Treg [37], [38] and their role in the control of Treg homeostasis in measles needs to be further analyzed. Finally, MV glycoproteins H and F have been shown to play the important role in T cell silencing by contact-mediated inhibition of cell proliferation [39], [40]. Although this mechanism may contribute to the immunosuppression in measles, the inhibitory role of MV glycoproteins was not found in the in vitro Treg assay in this study, probably due to the low level of cell membrane expression of glycoproteins, as shown in the figure 1A.

It is likely that measles-induced immunosuppression is not beneficial for the virus, as it does not prevent the generation of anti-viral immunity and virus clearance in a majority of patients. We postulate that the immunosuppression is generated as a consequence of the induction of the Treg system in host, in order to limit collateral tissue damage potentially provoked by a virus-induced vigorous activation of immune responses. This regulatory mechanism does not suppress an anti-viral reaction already initiated, as demonstrated clearly in measles [9], and therefore allows the generation of anti-measles immune response. However, it may prevent the initiation of new immune responses and consecutively, increase the susceptibility to opportunistic infections in patients. Thus, these results help in a better understanding of the immunological paradox associated with MV infection.

Measles is occasionally accompanied by the development of different forms of encephalitis, which is fatal in the majority of cases [2]. The reasons why an individual preferentially develops persistent MV brain infection after an acute infection, in spite of existing anti-viral immune response, are currently unknown. It has been suggested that a transient phase of immune suppression preceding MV infection may allow later development of neurological complications, like subacute sclerosing panencephalitis (SSPE) [41]. This transient immunosuppression following an acute infection, may also be due to MV-induced Tregs, which if induced to high levels, may favor the development of the viral neuropathology. It is thus tempting to speculate that the persistence of MV in the brain of some patients may be the price paid to limit brain immunopathology, induced by the immunomodulatory activity of Tregs recruited in the infected brain parenchyma. As Tregs could control persistent viral infections [40], [42], they may thus play a role in the establishment and maintenance of persistent MV infection in SSPE. Efficient manipulation of Treg cell population may therefore, be of critical importance in the prevention and treatment of measles-induced pathology.

## Materials and Methods

### Infection of mice

Heterozygous transgenic mice crossed in C57Bl/6 background and expressing human CD150 [24] and their littermate controls, as well as CD150 transgenic mice crossed into Foxp3-GFP background [29] or in IFN Receptor α/β deficient background [43] were bred at the institute's animal facility (PBES) and infected at the age of 1 week. Protocols were approved by the Regional ethical committee (CREEA). Mice were infected intranasally (i.n.) by application in both nares with 10 µl of wild type MV G954 [24] (from 200 to 1000 PFU) which gives clinical symptoms (ataxia, seizures, weight loss) in 75% of transgenic mice starting on 8 dpi. Control mice received the same amount of culture medium (RPMI 1640) intranasally.

### Cytofluorometric analysis

PerCP, FITC, PE and allophycocyanin-conjugated monoclonal antibodies to CD4, CD8α, CD25, CD62L, CD69, CD44, CD19, GITR and ICOS were purchased from BD Biosciences and eBioscience and MV hemagglutinin was stained using cl.55 mAb [44], [45]. Intracellular staining for Foxp3 was performed using APC anti-mouse Foxp3 staining set (eBioscience), for IFN-gamma, using PE anti-mouse IFN-γ (BD Bioscience) and for IL-10 using PE anti-mouse IL-10 (BD Biosciences), after treatment with PMA, ionomycine and brefeldine A. Cells were analyzed on a Facscalibur flow cytometer (Becton Dickinson).

### Determination of MV specific antibodies in serum

Sera were tested for anti-MV nucleoprotein (N) specific IgG antibodies by ELISA as previously described [24]. To determine neutralizing antibody titers, serum dilutions were incubated with 200 pfu of MV for 1 h at 37°C, and transferred into plates with confluent Vero-CD150 cell monolayers. The plates were read after 4 days by methylene blue staining and the dilution of serum reducing 50% of the virus was recorded. The titer of N-specific antibodies in each serum sample was determined using a standard curve established with sera from mice immunized with MV in complete Freund's adjuvant and expressed in relative units.

### MV N-specific CTL assay

Splenocytes from infected or non infected mice (10^7^ per well) were cultured in 24-well culture plates (Falcon) with Mitomycin C (Sigma) (40 µg/ml) treated-P815-N cells [46] (10^6^ per well) in RPMI 1640 medium, supplemented as described [24]. Viable lymphocytes were harvested 7 days later by density gradient medium using Lympholyte M (Cedarlane laboratories) and CTL assay was performed as described previously [47]. P815-N and P815 cells were labeled by a 13 min incubation at 37°C with 7.5 µmol CFSE per 2.10^7^ cells and 100 µl of various dilutions of effector cells suspensions were mixed with target cells, P815-N or P815 (2.10^4^ cells/100 µl). After 4 h incubation, cells were analyzed on a Facscan flow cytometer (Becton Dickinson). The percentage of dying cells among CFSE^+^ cells was determined using 0.4 µg/ml propidium iodide. The assay was performed in duplicate cultures and specific cytotoxicity was calculated by substracting the mean percentage of non-specific lysis obtained with P815 cells from the P815-N percentage of lysis.

### Immunohistofluorescence

Anesthetized mice were perfused with PBS. Brains were rapidly collected and snap-frozen in cold isopentane. Sections (10 µm) were fixed in ice-cold acetone, dried and blocked with 1% BSA/PBS. Biotin and avidin binding sites were blocked using Biotin/Avidin Kit (Vector Laboratories), before incubation with either rat anti-mouse CD4, rat anti-mouse CD8 (Serotec) or rat anti-mouse Foxp3 (eBioscience) and an anti-MV N protein mouse monoclonal Cl.120 biotinylated antibody overnight at 4°C. The specific labelling was revealed by rhodamine-conjugated streptavidin and a FITC-conjugated donkey anti-rat antibody (Jackson Immunoresearch) for 1 h at 37°C. Slides were viewed using a Axiovert 200 M microscope (Zeiss) and analyzed with the Axiovision software (Zeiss).

### Isolation of lymphocytes from the brain

Brains were harvested after perfusion with PBS. Lymphocytes were obtained from brain tissue as described previously [48]. Briefly, brains were diced, mashed and washed with DMEM and 7 ml of supernatant was mixed with 3 ml of 90% Percoll (in PBS; Amersham, Pharmacia Biotech) and layered on 1 ml of 70% Percoll (in DMEM). After a centrifugation at 1300 g, 30 min on 20°C, the interface was transferred to a new tube, washed and used for the immunostaining.

### In vitro suppression assay

CD4^+^ and CD4^+^CD25^+^ cells were purified using the isolation kits (Miltenyi Biotec) according to the manufacturer's recommendations. CD4^−^ cells were used as APC. The purity of all cell preparations was determined by flow cytometry. CD4^+^CD25^−^ T cells (2.10^4^ cells/well) were stimulated for 72 h with 1 µg/ml of Concavalin A (Sigma) in the presence of irradiated (1800 rad) CD4^−^ cell splenocytes (APCs) (10^5^/well) with indicated numbers of CD4^+^CD25^+^ T cells and pulsed with 1 µCi/well of [^3^H]thymidine for the final 12 h of culture. Data are shown as the mean percentage of inhibition of proliferation in triplicate cultures (+/−SD).

### Mixed leukocyte reaction (MLR)

Splenocytes harvested from uninfected or MV-infected transgenic and nontransgenic littermate mice (5×10^5^/well) were stimulated in MLR culture with the same number of irradiated (1800 rad) either allogeneic or syngeneic splenocytes obtained from either Balb/c or C57Bl/6 mice respectively, in complete RPMI medium. Cell proliferation was assessed on day 4 by [^3^H]thymidine incorporation for 18 h. Results are expressed as mean proliferation indices +/−SD ∶ (cpm of lymphocytes cultured with Balb/c or C57Bl/6 spleen cells)/(cpm of lymphocytes cultured alone).

### Assay for contact hypersensitivity (CHS) to DNFB

CHS to 1-fluoro-2,4-dinitrobenzene (DNFB) was determined as previously described [17]. Briefly, DNFB was diluted in acetone ∶ olive oil (4∶1) before use and 25 µl of 0.5% DNFB solution was applied to the shaved ventral skin (sensitization phase). After 5 days, mice received 10 µl of a non-irritant concentration of DNFB applied on both sides of the left ear and the solvent alone on the right ear (effector phase). Ear thickness was monitored before challenge and every day after challenge for three days, by a third experimenter ‘blinded’ to sample identity. The ear swelling was calculated as {[T-To ] left ear} - {[T-To right ear]}, where T and To are ear thickness after and before challenge, respectively.

### Statistical analysis

Data were expressed as mean+/−standard deviation (SD). Statistic analyses were performed using Student's t-test and Mann-Whitney U-test.
